# Factors affecting repurchase intentions in retail shopping: An empirical study

**DOI:** 10.1016/j.heliyon.2022.e10619

**Published:** 2022-09-13

**Authors:** Prodromos Chatzoglou, Dimitrios Chatzoudes, Athina Savvidou, Thomas Fotiadis, Pavlos Delias

**Affiliations:** a Democritus University of Thrace, Greece; b International Hellenic University, Kavala University Campus, Greece

**Keywords:** Retail stores, Shopping experience, Customer experience, Customer satisfaction, Customer loyalty, Repurchase intention

## Abstract

The present study investigates the factors affecting consumer repurchase intentions in retail stores. More specifically, it emphasizes on the concept of in-store customer shopping experience. In that direction, a new conceptual framework (research model) is developed and empirically tested, using primary data collected from retail store customers. The proposed model includes twelve research factors that are classified into three dimensions (groups): six independent factors (antecedents), five mediating factors and repurchase intention (dependent factor). In more detail, the study examines the antecedents of customer behavior, which constitute the in-store customer shopping experience (Physical environment, Interior shop environment & layout, Interaction with the staff, Interaction with other customers, Merchandise value/quality, Merchandise variety). It argues that the effect of the antecedents on repurchase intention is indirect, mediated through five other factors (mediators) (Customer experience, In-shop emotions, Perceived value, Customer satisfaction, Customer loyalty). Under that context, eleven research hypotheses were tested, using the Structural Equation Modeling (SEM) technique. The final sample includes 618 retail store customers, who participated in a web-survey. Results offer support for the underling mechanism of the proposed research model, arguing that antecedents significantly affect the mediators, which, in turn, affect the repurchase intention of retail shoppers. Results indicate that in order to have more return customers, retailers should enhance their interior shop environment and layout and increase the value of their merchandise. The originality of the study lies in its three-dimensional approach. It offers an understanding about the mechanism that impacts repurchase intentions, an approach lacking in the relevant literature. Moreover, it focuses on all kinds of retail stores, offering wider generalizability of its empirical findings. Also, it examines in-store emotions and experience of customers inside a store, two factors which very seldomly have been investigated in the context of physical retail stores.

## 1 Introduction

Customer purchasing behavior (i.e., trends, culture, and even a consumer's way of life) is an aspect of human behavior that is expressed as a set of processes relating to the interaction between the individual and the environment (Orji, 2013).

Customers are influenced by all attributes of a shop, from the search for products to everything that may follow the actual purchase (Yang et al., 2012; Mouri et al., 2015; Terblanche, 2018). In this respect, customer buying experience (CBE) is a notion that refers not only to the overall experience of customers while they are completing a purchase but, also, to the set of processes that precede and follow it (Otieno et al., 2005). Meyer and Schwager (2007) defined customer experience as an internal and subjective response during the process of directly coming to contact with the company - a definition also adapted from Faizan et al. (2016).

It is important to understand which factors play the most significant role, as well as to reveal the way they interact and the way they could guide customer buying behavior. This constitutes a complex problem with its parameters continuously changing over time. Usually, these factors are divided into intangible factors (e.g., the kind of interaction between consumer-staff or between consumers) and tangible factors (physical setting of the store, lay-out, merchandise etc.).

This paper focuses on the shopping experience inside a retail store. More specifically, it examines which store characteristics, both tangible and intangible, have an influence on various aspects of consumer behavior. The proposed model is based on a synthesis of previous concepts, theories and empirical studies. It investigates future repurchase intentions of retail store customers. In doing so, it develops a three-dimensional research model, including twelve factors. In more detail, the proposed research model examines the direct impact of six antecedents (store characteristics) on five aspects of consumer behavior. The study posits that these five factors mediate the impact of the antecedents on the repurchase intention of customers. The aim of the statistical analysis is to offer a model that eventually reflects what drives consumer behavior and repurchase intention.

Section 2 discusses the relevant literature and presents the main contribution of several related works. Section 3 is dedicated to explaining the factors and the hypotheses that were developed, while in Section 4 the research methodology is presented. The results are presented in Section 5, followed by an additional section dedicated to their discussion. Finally, the paper is concluded by providing an overview of the findings and by discussing their implications.

## 2 Previous studies

According to Oliver (1997) “*... Everybody knows what satisfaction is, until they are asked to define it... Then it appears that nobody knows...*“. Satisfaction has been considered a partly cognitive, party emotional evaluation of a customer's experience of service settings (Oliver, 1997; Westbrook and Oliver, 1991; Wirtz and Bateson, 1999). Emotion and knowledge are two important elements of satisfaction (Ali et al., 2016; Kollman, 2000), especially in the context of modeling consumer behavior in a service setting (Wong, 2004). Furthermore, Parker and Mathews (2001) argued that satisfaction is not only related to the concept of a product, but also to all the accompanying services provided.

Customer satisfaction is the primary goal of any enterprise, due to its potential impact on repeated purchasing behavior and increased profits (Kim and Brymer, 2016; Ryu et al., 2012). Johnson et al. (2001) stressed out that the cumulative satisfaction of customers tends to be a good forecast of their future behavior. Marting et al. (2008) observed that customer satisfaction based on emotions (as such stemming from various experiences) is a more potent prognostic indicator for future behavior intentions. In the same line, Hart et al. (2007) suggested that the quality of a purchase experience has a positive impact on customers’ intention to repurchase, while a pleasant experience is also connected to their increased support. Chan et al. (2015) considered support intentions (comprised of two dimensions: purchase intentions and future preference potential) as the only measure able to forecast future customer behavior. Similarly, Bolton et al. (2000) claimed that customer repurchase intentions depend on prior positive behaviors and their satisfaction over the passage of time.

Repurchase intention and the continuous support of a store by customers are interwoven with their satisfaction, so the concepts of customer loyalty, repurchase intention and future support are closely related to one another. Actually, cumulative customer satisfaction was found to provide the highest forecast of loyalty (Johnson et al., 2001; Bodet, 2008; Nam et al., 2011), while other researchers (Ruffin et al., 2012; Lin and Lin, 2011) found that loyalty intentions derive from customer satisfaction. In addition, various studies supported the fact that satisfaction directly influences repurchase intention, as well as the purchasing behavior of a customer (Kumar, 2002; Mittal and Kamakura, 2001).

Loyal customers do not pay much attention to price (Jaiswal and Niraj, 2011) and purchase additional goods and services offered by the same business to which they are loyal. They also appear to need less assistance, thanks to their familiarization with the provider and tend to be more efficient with respect to the use of the company's resources (Duffy, 2003), Delias and Matsatsinis (2009), Sharma et al. (2009) and Zhang et al. (2018) found out that customer loyalty is correlated with the quality of decision making. Further, loyal customers are more resilient to possible bad experiences and defective products and prefer to complain without stirring more hassle, offering the company a second chance (Duffy, 2003). In addition, customer loyalty can increase the market share and sales of a company and improve its competitive advantage (Agrawal et al., 2012).

De Keyser et al. (2015) defined Customer Buying Experience (CBE) as the holistic experience comprising of cognitive, emotional, social and physical elements. There is a direct association between consumer experience and satisfaction. Customer satisfaction is not only considered to be an effect of the actual purchase, but also a result of the services the client enjoys during and after the buying process (Terblanche, 2018). Ali et al. (2016) investigated how customer experience is affected by the physical setting of the premises, the interaction with the staff and other customers. They showed that all these factors have a significant impact on customer euphoria and satisfaction.

Walsh et al. (2011) showed that knowledge about the store affects emotions, satisfaction and customer loyalty in different ways, while euphoria and pleasure mediate the relationship between the cognitive function that is related to the store and customer outflow.

Customer satisfaction and perceived value are the most influential factors forecasting the level of customer trust (Yang et al., 2004). Terblanche (2018) revealed that merchandise variety, interaction with the staff, the ambiance inside the store and the emotions evoked while being in the shop all have a strong positive cumulative influence on customer satisfaction, which is, in turn, associated with repurchase intention.

In addition, customer satisfaction was found to be closely related to perceived value (e.g., Cronin et al., 2000; Bajs, 2015). Perceived value constitutes a psychological evaluation of a product or service that is based on the subjective evaluation of the consumer (Tynan et al., 2010). Fornell et al. (1996) emphasized the concept of perceived value, advocating that quality is the first determinative factor of total customer satisfaction, while the second is value. Satisfaction, therefore, results from perceived value. Perceived value differs from satisfaction, thus customer preferences for services and their purchase intentions can be measured by examining their perceived value.

Finally, Mason and Himes (1973) argue that satisfaction is directly related to demographic and socio-economic factors (e.g., education, affluence, age, and maturity).

A brief presentation of some of the relatively recent studies of this area is presented in Table 1.

**Table 1:** Relevant research (indicative list).

Empirical research	Methodology	Sample	Factors affecting consumer buying behaviors	Method of Hypothesis testing
Zhang et al., 2018	Survey/Use of Structured Questionnaire	528 customers of a Chinese Store (Mogujie)	Online product recommendations, Perceived decision effort (Product screening cost, Product evaluation cost), Perceived decision quality (Dependent factor: Customer loyalty)	Partial Least Squares Structural Equation Modeling (PLS-SEM)
Karatepe (2006)	Survey/Use of Structured Questionnaire	781 hotel customers in a holiday destination	Complainant satisfaction, Distributive justice, Procedural justice, Interactional justice (Dependent factor: Customer loyalty)	Covariance-based Structural Equation Modeling (CB-SEM)
Yang and Peterson (2004)	Survey/Use of Structured Questionnaire	235 internet users	Customer value, Perceived satisfaction, Switching cost (Dependent factor: Customer loyalty)	CB-SEM and Hierarchical moderated regression analysis
Terblanche (2018)	Survey/Use of Structured Questionnaire	329 supermarket customers	Merchandise value, Merchandise variety, In-shop emotions, Internal shop environment, Interaction with staff, Interaction with other customers (Dependent factor: Customer satisfaction), Customer satisfaction (Dependent factor: Intention to repatronage)	CB-SEM
Ali et al. (2016)	Survey/Use of Structured Questionnaire	292 visitors of thematic parks (Kuala Lumpur and Selangor)	Physical environment, Interaction with the staff, interaction with customers, Customer delight (Dependent factor: Customer Satisfaction), Customer delight, Customer satisfaction (Dependent factor: Customer loyalty)	PLS-SEM
Chen and Lin (2015)	Survey/Use of Structured Questionnaire	452 social media users	Customer Experience, Perceived Value (Dependent factor: Satisfaction), Customer Experience, Perceived Value, Satisfaction (Dependent factor: Continuance Intention)	PLS-SEM
Wu and Li (2018)	Survey/Use of Structured Questionnaire	599 Facebook users	Customer value in social commerce (Utilitarian value, Hedonic value, Social value) (Dependent factor: Customer loyalty in social commerce)	PLS-SEM
Syahrial et al. (2017)	Survey/Use of Structured Questionnaire	310 Japanese who owns a car or an air-condition	Tangibles, Assurance, Responsiveness, After-sales service cost (Dependent factor: Customer Satisfaction/Loyalty)	CB-SEM
Lee et al. (2008)	Survey/Use of Structured Questionnaire	472 visitors of the International Dance Festival of Andong	Festival environment (Dependent factors: Positive emotion, Satisfaction, Negative emotion), Positive emotion, Satisfaction, Negative emotion (Dependent factor: Loyalty)	CB-SEM
Walsh et al. (2011)	Survey/Use of Structured Questionnaire	274 customers of four coffee making shops of the same chain	Store related cognitions (Dependent factors: Arousal, Pleasure, Store satisfaction), Arousal, Pleasure, Store satisfaction (Dependent factor: Store loyalty)	CB-SEM

Based on the results of the literature review analysis, some gaps/limitations of this particular research field were discovered. Initially, it seems that most of the existing studies focus on specific sectors (hotels, theme parks, festivals and super-markets) something that sets obstacles and limits the generalization of their conclusions to other sectors of the economy.

Many of these studies (see Table 1) relied on the users of one networking medium, a particular (electronic) shop or one ethnic group. The size of the samples used were rather small, while in the majority of these studies there was a rather limited measurement of emotions with a negative impact on buying behavior. Finally, according to Zhang et al. (2018), several of these studies seem to be biased, since many authors reference their own work and built their frameworks upon their previous empirical attempts.

The present study is built in order to bridge these gaps in the relevant literature. In that direction, it does the following: (a) It focuses on all kinds of retail stores, offering wider generalizability of its results; (b) In that vein, it offers consumers the chance to select the retail store they frequently visited during the last six months prior their participation in the survey, thus avoiding to limit its sample on a preselected store, something which is the case for most previous studies of the field (e.g., Lee et al., 2008; Walsh et al., 2011), (c) It includes a sufficient sample (n = 618), increasing the validity of its results (most previous relevant studies use smaller samples, see Table 1); (d) the development of its conceptual framework is based on a synthesis of numerous theories and models (e.g., utility theory, environmental psychology theory, social-psychological model), an approach also not explicitly followed by many previous studies (e.g., Ali et al., 2016; Karatepe, 2006). These theories and models are analytically discussed in Section 3.

Despite the fact that consumer repurchase intentions have already been studied in numerous prior papers (e.g., Terblanche, 2018; Chen and Lin, 2015), the present study is one of the few to introduce the following aspects: (a) It incorporates a set of independent factors (antecedents; aspects of in-store customer shopping experience) that predict the variance in customer behavior. These (six) factors have very rarely been used by previous researchers. Even the few studies that used them (e.g., Terblanche, 2018), failed to examine their indirect effect on final outcomes, like repurchase intentions. Future studies can use the same three-dimensional approach (antecedents → mediators → repurchase intention); (b) It examines the role of “in-shop emotions” from an environmental psychology perspective and explores their role in translating (mediating) the impact of in-store attributes into actual outcomes, like repurchase intentions. Very few studies (e.g., Terblanche, 2018; Han and Hyun, 2018) have incorporated that same factor and the same approach in their analysis.

In-shop emotions represent the emotional response of customers while visiting a physical store. In that context, Han and Hyun (2018) consider in-shop emotions as affective responses to the actual shopping experience. Numerous previous studies in consumer behavior and marketing found that emotions significantly influence customer satisfaction and behavioral (repurchase) intentions (e.g., Dubé and Menon, 2000; Han, 2014; Terblanche, 2018; Westbrook, 1987). According to Terblanche (2018), positive emotional experiences within a store increase shopping satisfaction and, finally, enhance repatronage intention. Moreover, Oh (2005) found that (positive and negative) emotions are determinants of customer behavioral intentions. In the present study, it would be interesting to further examine the role of in-shop emotions and determine its antecedents and effects.

## 3 Research model and hypotheses

The research model proposed in this study has its roots in the relevant literature of the field. On a wide perspective, it is based on the “consumer behavior theory” (Kotler and Armstrong, 2011), which examines how consumers make purchasing decisions. According to Mou and Benyoucef (2021), consumer behavior is highly important, since it examines the rationale behind consumer actions and preferences; it answers to what, where, how, when and why consumers prefer certain goods (or retail stores) versus others (Buerke et al., 2017).

Many specific theories and models have been used in order to predict consumer behavior. These include (Mullen and Johnson, 2013): economic theories (e.g., marginal utility theory), psychological theories (e.g., cognitive theory), social psychological theories (e.g., cognitive dissonance theory), and sociological theories (e.g., role theory). This paper deals with the in-store customer shopping experience. Under that context, it examines which store characteristics (independent factors) have an influence on various aspects of consumer behavior. The six antecedents of consumer behavior used in this study are adopted from three prominent theories of the field.

“Physical environment” and “Interior shop environment & layout” fall into the environmental psychology theory (Mehrabian and Russell, 1974a, Mehrabian and Russell, 1974b), which argues that a store's atmosphere has an influence on the mood and behavioral intentions of customers (Michon et al., 2008). Pleasant surroundings are supposed to make customers want to stay longer, explore the store, and affiliate with other shoppers and/or salespersons (Donovan and Rossiter, 1982). According to Lee and Jeong (2012), all physical and tangible cues of a store can have an effect on the emotional state and the decision-making process of consumers.

“Interaction with the staff” and “Interaction with other customers” have their roots in the Veblenian social-psychological model of human behavior. According to that theory, as it has been adopted by the consumer behavior domain, customers are social beings and their buying behavior is influenced by their immediate social counterparts (Patsiaouras and Fitchett, 2012). The present study posits that in a retail store, these counterparts are the employees and the fellow customers, with which the customer interacts.

“Merchandise value (quality)” and “Merchandise variety” are theoretically based on the utility theory. According to Jarvis et al. (2017), consumer behavior depends on the need of consumers to buy products with desirable attributes. Under that theory, consumers are quite reasonable and purchase goods (and services) in order to get certain benefits. When a store has products with high quality (and/or value) and offers many choices to its customers, it satisfies their utilitarian motives (Martín-Consuegra et al., 2019).

**Figure 1:**
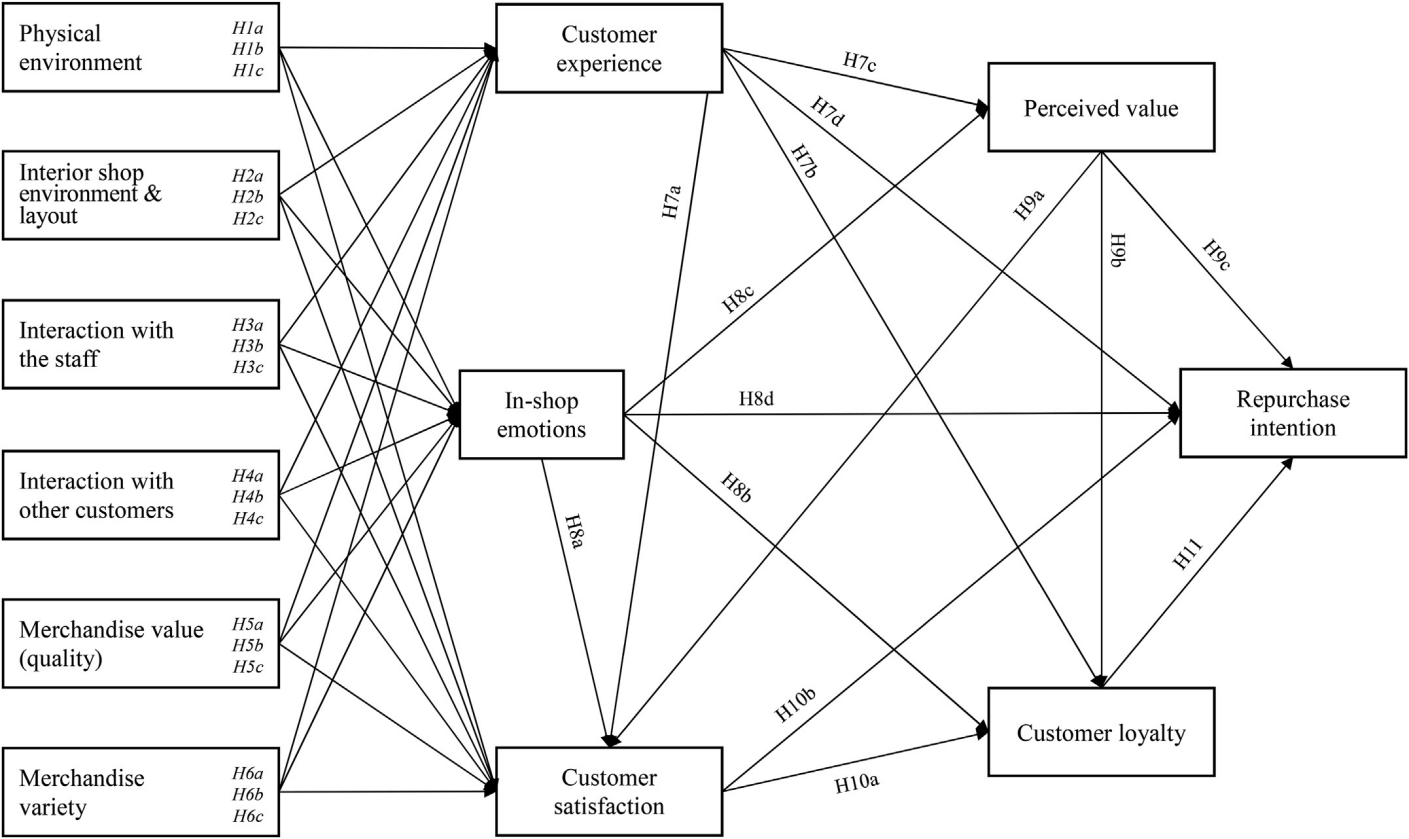
The proposed conceptual framework of this study.

The proposed research model of this study (depicted in Figure 1) examines the direct impact of the six antecedents described above, on five aspects of consumer behavior (customer experience, in-shop emotions, perceived value, customer satisfaction, customer loyalty). These five factors are hypothesized to mediate the impact of the six antecedents (or independent factors) on the repurchase intention of customers (which is the main dependent factor of this study). Hence, their role is mediating.

As such, the present study introduces an original conceptual framework (research model) that includes twelve (12) research factors. These factors are classified into three groups (dimensions), according to the role they play in affecting the repurchase intention of consumers:

A) Six independent factors (capturing the “in-store customer shopping experience”): Physical environment, Interior shop environment & layout, Interaction with the staff, Interaction with other customers, Merchandise value (quality), Merchandise variety.
B) Five mediating factors (capturing various aspects of consumer behavior): Customer experience, In-shop emotions, Perceived value, Customer satisfaction, Customer loyalty.
C) One outcome (dependent) factor: Repurchase intention.

The proposed model integrates several relevant concepts and theories into a common framework that investigates future repurchase intentions of retail store customers. Its development is based on an extensive literature review. Initially, a review of the theory and empirical literature relating to in-store customer shopping experience was conducted. Table 1 presents a synopsis of that effort. From that process, the antecedent (independent) and mediating factors were recognized. As mentioned above, antecedents stem from three different theoretical approaches. Mediators are selected on the basis of their significance. The review of the empirical literature highlighted that these five factors (mediators) have emerged as the most influential in studies that are concerned with physical retail stores (Ali et al., 2016; Terblanche, 2018; Zhang et al., 2018).

Customer satisfaction and customer loyalty are widely used concepts in behavioral intention studies. For example, Ryu and Han (2010) examined the role of customer satisfaction, as a mediating factor, in the relationship between quality dimensions and customer behaviors in quick-casual restaurants. Customer loyalty has also been frequently examined, mostly as a dependent factor (e.g., Bouzaabia et al., 2013; Thomas, 2013). Despite that, the present study argues that loyalty has a significant mediating role, translating the effects of in-store experience in actual behavioral intentions (like repurchase intention).

Moreover, customer experience is a very contemporary concept. According to Becker and Jaakkola (2020), it has received significant attention in both marketing research and practice during the last ten years. This is because business leaders perceive customer experience as a main prerequisite to competitiveness, while academics argue that it is the fundamental basis for marketing management (Lemon and Verhoef, 2016). Customer experience is very important in a store setting, since it builds the basis for future purchasing interactions (Bueno et al., 2019).

Finally, in-shop emotions and perceived value were incorporated into the proposed conceptual framework. According to Chen and Lin (2015), perceived value, the tradeoff between perceived benefits and perceived costs, is able to accurately explain purchase decisions, and is a major factor that can differentiate companies and assist in maintaining a competitive advantage. In-shop emotions have rarely been examined in previous studies. Since the present study examines the effects of the overall in-store shopping experience, the inclusion of in-shop emotions seems like a reasonable choice; it would be interesting to examine how various aspects of the shopping experience affect the levels of happiness, excitement and enjoyment a customer feels when being inside a retail store. It would, also, be interesting to examine whether these feeling impact future buying decisions.

### 3.1 Physical environment

Physical environment describes the tangible features of an undefined service or set of services. It includes various elements, such as color, air, scents, illumination, installations and layout (Han and Ryu, 2009; Lin and Liang, 2011; Ali et al., 2016). These factors are coexisting and intertwined (Mehrabian and Russell, 1974a, Mehrabian and Russell, 1974b) in order to influence enjoyment, satisfaction and consumer behavior from different perspectives (Ariffin and Yahaya, 2013). Numerous surveys demonstrated, in detail, that physical environment can cause both positive and negative emotions and affect customer satisfaction (Burns and Neisner, 2006; Ladhari, 2009; bib_Kim_et_al_2009J. Kim et al., 2009; Pareigis et al., 2011). Nevertheless, there is a need for an even more thorough investigation of its effectiveness, especially on customer satisfaction (Ali et al., 2016). Thus, the following set of hypotheses is proposed:

H1a The quality of the Physical environment enhances In-Shop Emotions.

H1b The quality of the Physical environment enhances Customer Experience.

H1c The quality of the Physical environment enhances Customer Satisfaction.

### 3.2 Interior shop environment & layout

The interior shop environment refers to the decoration of the store, as well as the physical installations and infrastructure, the layout of the floor, the manner by which products and services are grouped, the allocation of products on the shelves, the place and their role in encouraging purchases (et al. (2013) stressed the importance of interior layout. According to Mohan et al. (2012), an Terblanche, 2018). Marques efficient and effective layout can facilitate customers to explore the premises, thus positively affecting their satisfaction. As Morales et al. (2005) claim, “proper” interior planning not only drives customers to making purchases, but also boosts their level of satisfaction stemming from product selection. According to Lichtlé and Plichon (2014), various studies focused on what is known as the “kinetic quality” of a shop's environment -the movements and gestures of customers during purchases-which further reinforces their purchasing experience and satisfaction (Bonnin and Goudey, 2012; Borghini et al., 2020). The following hypotheses are formulated:

H2a An effective and efficient Interior shop environment & layout enhances In-Shop Emotions.

H2b An effective and efficient Interior shop environment & layout enhances Customer Experience.

H2c An effective and efficient Interior shop environment & layout enhances Customer Satisfaction.

### 3.3 Interaction with the staff

The interaction between customers and staff, as well as the extend that this interaction might influence the emotional attachment and the total customer experience have been examined by many studies (Slatten et al., 2011; Verhoef et al., 2009; Wallsetal, 2011). It is widely recognized that the interaction between customers and employees affects the purchasing experience in the shop (Brown and Lam, 2008), while customer satisfaction is also frequently influenced by the quality of the interpersonal interaction between customers and employees (Menon et al., 2000; Puccinelli et al., 2009). Pan and Zinkham (2006) argued that the friendliness and know-how of the sales staff are important factors for a shop's future attractiveness. Further, Marques et al. (2013) showed that personnel assistance is the second most important factor leading to customer satisfaction and is related with the stimuli customers get from a shop. In the same line, Zeithaml et al. (2006) claim that things like a smile or small details, like a pleasant voice or a friendly disposition by staff members, may influence customer emotions, perceptions and their total experience (Slatten et al., 2011). Thus, the following hypotheses are proposed:

H3a Effective and efficient Interaction with the staff enhances In-Shop Emotions.

H3b Effective and efficient Interaction with the staff enhances Customer Experience.

H3c Effective and efficient Interaction with the staff enhances Customer Satisfaction.

### 3.4 Interaction with other customers

Buying experience inside a store is basically a social activity, which is also affected by the interaction with other customers (Terblanche, 2018). Brocato et al. (2012) investigated the influence of other customers on the assessment of buying experience. Pons et al. (2016) noted that other customers can contribute to a pleasant experience and even bring about a positive effect for the enterprise itself. Tombs and McColl-Kennedy (2003) found that verbal interaction between customers frequently assumes a complementary or substitute role to staff's efforts to conclude a sale and may significantly affect customer satisfaction and their perceptions about the quality of the provided services. Interestingly, when customers feel that they are useful to other customers, they feel more satisfied from their purchasing experience inside a shop (López-López et al., 2014). The following hypotheses are, thus, formulated:

H4a Fulfilling Interaction with other customers enhances In-Shop Emotions.

H4b Fulfilling Interaction with other customers enhances Customer Experience.

H4c Fulfilling Interaction with other customers enhances Customer Satisfaction.

### 3.5 Merchandise value (quality)

Baker et al. (2002) defined merchandise value as the result of the price of the merchandise and its quality. As such, merchandise value significantly affects repurchase intention. Sirohi et al. (1998) suggested that merchandise value is a compromise between the money spend and the benefits offered by a store (Terblanche, 2018). Cronin et al. (2000) noted that customer satisfaction is a result of the quality and value of the merchandise. Previous studies have found that customer perceptions about merchandise quality influence their satisfaction and loyalty (e.g., Walsh et al., 2011). Similarly, Poncin and Mimoun (2014) stated that merchandise value positively affects satisfaction (Carpenter and Moore, 2009; Shobeiri et al., 2013), while Sivadas and Jindal (2017) also agreed that merchandise value heavily affects customer satisfaction. Based on the above, the following hypotheses are proposed:

H5a Merchandise value enhances In-Shop Emotions.

H5b Merchandise value enhances Customer Experience.

H5c Merchandise value enhances Customer Satisfaction.

### 3.6 Merchandise variety

Merchandise variety is defined as the cognitive factor that leads to positive customer behavior. According to Pan and Zinkhan (2006), it is the most important factor for customers when they rank their preferences and select a store. A similar relationship was found by Morales et al. (2005), Bauer et al. (2012) and Marques et al. (2013). More specifically, Morales et al. (2005) argued that consumer decisions are positively associated with merchandise variety, while Marques et al. (2013) concluded that merchandise variety is the factor with the greatest impact on customer satisfaction. Thus, the following hypotheses arise:

H6a Merchandise variety enhances In-Shop Emotions.

H6b Merchandise variety enhances Customer Experience.

H6c Merchandise variety enhances Customer Satisfaction.

### 3.7 Customer experience

Customer experience is defined as the cognitive, emotional and behavioral response of a customer, who observes or participates in an event, while inside a store (Godovykh and Tasci, 2020; Jain et al., 2017). Schmitt (1999) suggested five types of experience, which could form the basis for a total experiential analysis, namely: sensing, feeling, thinking, acting and relating. Customer experience constitutes the psychological perception in the users’ hearts and considerably influences future behavior (Hsu and Tsou, 2011).

Customer experience has been found to be associated with satisfaction (Torres et al., 2014). When customer experience is enhanced, consumers have positive feelings and feel satisfied (Kim, 2005; Koufaris, 2002). Mascarenhas et al. (2006) argued that customer experience is the contemporary differentiator between companies and that loyalty depends upon the experiences customers have at any given point. The concept of experience takes under consideration the emotional responses of customers and goes beyond product satisfaction. Srivastava and Kaul (2016) found that building loyalty is based on the successful management of customer experiences.

The relationship between customer experience and perceived value has not been previously discussed in the literature. Perceived value captures the understanding of customers about the overall value they receive from a store, in comparison with other competitors. Value is perceived in terms of cost, value for money and service provision. This study argues that when customers have a positive emotional disposition towards a store (enhanced experience), they tend to perceive that more value is being received. In other words, positive emotional responses make consumers move beyond a rational evaluation of a store and increase their understanding about the value of its offerings.

Concerning the relationship between customer experience and repurchase intention, most previous studies have been conducted in an online environment (e.g., Amoako et al., 2021; Anshu et al., 2022; Liu et al., 2016). According to Cowley (2008), past experience provides customers with the ammunition to rationalize a desired activity. Under that context, the past has been found to justify the future. Customers tend to repeat a purchasing behavior when their past experience is positive (Anshu et al., 2022; Martin et al., 2015; Li et al., 2022).

H7a The improvement of Customer Experience enhances Customer Satisfaction.

H7b The improvement of Customer Experience enhances Customer Loyalty.

H7c The improvement of Customer Experience enhances Perceived Value.

H7d The improvement of Customer Experience enhances Repurchase Intention.

### 3.8 In-shop emotions

In-shop Emotions are defined as the emotional state of consumers that directly affects their satisfaction and behavior. Burns and Neisner (2006) found that the level of customer satisfaction can be explained in terms of both cognitive evaluation and emotional reaction. Donovan and Rossiter (1982) concluded that customers’ emotions, while they are in the store, influence the potential for future purchases. Dawson et al. (1990) believe that in-shop emotions influence customer preferences and product selection, while positive emotions may also affect customer satisfaction. Similarly, Burns and Neisner (2006) verified that in a purchasing environment, emotions play an important role for customer satisfaction. Furthermore, Machleit and Mantel (2001) suggest that evoked emotions that are attributed to a store exercise a more forceful effect on customer satisfaction, than those attributed to external factors. Finally, Han and Hyun (2018) established the significant role of (positive and negative) in-shop emotions in affecting customer behavioral intentions towards airport duty-free shops.

H8a Positive In-shop Emotions enhance Customer Satisfaction.

H8b Positive In-shop Emotions enhance Customer Loyalty.

H8c Positive In-shop Emotions enhance Perceived Value.

H8d Positive In-shop Emotions enhance Repurchase Intention.

### 3.9 Perceived value

Perceived value is understood as the ratio of the benefits received by customers in relation to the costs associated with a purchase (Woodruff, 1997). In this study, perceived value is measured in terms of the cost, value for money and services provided by a retail store, in comparison with other competitors. The concept of value has received much attention by marketers during the last decade, since customers become more and more value-driven (El-Adly, 2019).

Since perceived (retail store) value has not been thoroughly investigated before, the following three hypotheses are based on general theoretical observations. Firstly, when customers feel that their purchase has significant value, they tend to be satisfied (Anderson and Mittal, 2000; Yang and Peterson, 2004). It seems reasonable that getting more value from a purchase will increase the levels of satisfaction with a retail store (Ryu et al., 2012). Secondly, when customers receive added value from an interaction with a store, they tend to increase their levels of loyalty. This is especially the case when increased value is consistently received on a continuing basis (Tsai et al., 2010). Under that context, value-seeking customers will be loyal to establishments that satisfy their need to receive additional value from their purchases. Thirdly, customers that perceive that a retail store offers additional value, will tend to return for additional purchases. According to Kim et al. (2012), this is the case for both utilitarian and hedonic shopping. Marketing psychology suggests that customers wish to repeat an experience that offered them specific benefits in the past (Chiu et al., 2014).

Therefore, it would be interesting to examine the following set of hypotheses, since most of them have not been thoroughly investigated by the relevant empirical literature:

H9a Perceived value enhances Customer Satisfaction.

H9b Perceived value enhances Customer Loyalty.

H9c Perceived value enhances Repurchase Intention.

### 3.10 Customer satisfaction

Customer satisfaction is the partially cognitive and partially emotional evaluation of the (shopping) experience (Oliver, 1997; Westbrook and Oliver, 1991; Wirtz and Bateson, 1999). Westbrook and Oliver (1991) simply defined customer satisfaction as the assessment of a specific transaction (Ali et al., 2016).

Various researchers suggested that customer satisfaction constitutes a factor of huge importance for building customer loyalty (Anderson et al., 2004; Homburg et al., 2006; Loureiro, 2010). Empirical results have shown that the more satisfied customers are, the more loyal they will be and the more purchases they will conduct in the future. The literature provides evidence that customer satisfaction constitutes a significant antecedent of loyalty (Johnson et al., 2001; Bodet, 2008; Nam et al., 2011). Similarly, Chitty et al. (2007) concluded that satisfaction with respect to the rendered services can lead to loyal customers, while Kao et al. (2008) verified the impact of customer satisfaction on customer loyalty. In addition, Jones and Reynolds (2006) found that customer satisfaction from a store constitutes a significant predictive factor of repurchase intentions, while López-López et al. (2014) verified the positive correlation between customer satisfaction and repurchase intention. Finally, various other researchers consider repurchase intention as a consequence of customer satisfaction (Rufín et al., 2012; Lin and Lin, 2011).

H10a Customer satisfaction enhances Customer Loyalty.

H10b Customer satisfaction enhances Repurchase Intention.

### 3.11 Customer loyalty

Customer loyalty is related to various consumer behaviors, such as repurchase intention, word of mouth communication, as well as the recommendation of the company to others (Karatepe, 2006), while it is also found to have a significant effect on behavior and consumer attitude (Gonçalves and Sampaio, 2012; Kato, 2019; Shkoukani et al., 2017). Thus, the following hypothesis emerges:

H11 Customer loyalty enhances Repurchase Intention.

Figure 1 illustrates the proposed conceptual framework of this study that focuses on the causal relationships between the various research factors.

## 4 Research methodology

This study is empirical (it is based on the collection and analysis of primary data), explanatory (examines causal relationships between twelve factors), deductive (develops and tests eleven research hypotheses) and quantitative (analyzes quantitative data collected with the use of a structured questionnaire).

### 4.1 Criteria for participating in the survey

For collecting the necessary primary data, an online survey questionnaire was used (web-based survey approach). The population of this study consists of Greek citizens between their 18^th^ and 60^th^ year of age. Every single responded was asked to provide their consent explicitly and electronically in order to participate to the study. The study focuses on retail stores. Therefore, respondents were asked to select a store they often visited during the last six months prior to their participation in the survey. All questions measuring the twelve factors of this study had to do with that specific store. In the first section of the survey, respondents were asked to write down the store they had in mind. They were analytically instructed that all their answers should concern that specific retail store. The same approach has been used in previous studies (e.g., Kim et al., 2009). Those who reported no frequent visits in any store during the last six months were disqualified from the data collection process. The same applied for people beyond the age limit. Additionally, participants were asked to select retail stores that, according to their understanding, employ more than five (5) employees. Hence, quite small shops were excluded from the sample. In plain words, the web-survey used appropriate filtering questions in order to ensure the participation of only the right respondents. Respondents not meeting the desirable criteria were not included in the final sample of the study.

### 4.2 Measurement

Each factor was measured using a number of variables (items) that were adopted from previous empirical studies (e.g., Ali et al., 2016; Chen and Lin, 2015; Syahrial et al., 2017; Terblanche, 2018; Yang and Peterson, 2004). Table 2 presents the research factors, the number of items used to measure each factor and the sources they were adopted from. Moreover, Appendix 1 includes all the items used for the measurement of the research factors.

**Table 2:** Measurement of the research factors.

Factors	Sources	Number of Items
Physical environment	Ali et al. (2016)	5
Interior shop environment & layout	Terblanche (2018)	4
Interaction with the staff	Ali et al. (2016); Terblanche (2018)	4
Interaction with other customers	Ali et al. (2016); Terblanche (2018)	4
Merchandise value (quality)	Terblanche (2018)	4
Merchandise variety	Terblanche (2018)	4
Customer experience	Chen and Lin (2015)	8
In-shop emotions	Lee et al. (2008)	6
Perceived value	Yang and Peterson (2004)	4
Customer satisfaction	Ali et al. (2016); Terblanche (2018)	4
Customer loyalty	Zhang et al. (2018); Wu and Li (2018)	4
Repurchase intention	Terblanche (2018)	4
**Total**		**55**

All items were measured using a conceptual (subjective) scale (5-point Likert scale). Participants could omit questions that were not willing, for whatever reason, to complete. In plain words, there were no restrictions imposed during the design of the questionnaire, mandating participants to complete any specific question. Despite that, questionnaires that included missing values in all the items of any given factor were discarded from the statistical analysis (they were deleted from the database by the researchers, after an initial review). Moreover, questionnaires that included more than 6 missing values in a total of 55 items used to measure the twelve factors of the study were also discarded. Missing values were not a real concern for the validity of this study. This is because the examination of the proposed research model was based on mean factor scores, and these scores included no missing values for all 618 valid questionnaires used in the statistical analysis.

The appropriateness of all items and their proper understanding from the target population (due to potential translation issues) were checked. For example, the back-translation method was employed; all items were translated back to the original language to secure its correspondence with the original version and its adjustment to the Greek reality.

The final form of the questionnaire included two sections. The first included several questions concerning the basic demographic characteristics of each participant, while the second section included 55 items employed to measure the factors of the proposed research model.

### 4.3 Data collection

As mentioned earlier, for collecting the appropriate empirical data for this study, a web-survey was conducted. The corresponding link was posted in various social media sites, following a rather popular practice in the field of empirical studies that examine buying behaviors and customer loyalty (e.g., Zhang et al., 2018; Yang et al., 2004; Chen and Lin, 2015; Wu and Li, 2018; Ali et al., 2016; Terblanche, 2018; Syahrial et al., 2017; Karatepe, 2006). Besides that, the use of the internet for collecting primary data is deemed as a legitimate and tested practice, in cases where internet users constitute a significant segment of the survey population (Belanche et al., 2012).

The web-based questionnaire was developed and distributed using Google services. In its first section, it described the main research purpose and underlined that all collected data are strictly confidential. It was posted at various popular internet sites (e.g., Facebook, Twitter, LinkedIn, etc.) in order to target different categories of users (citizens/buyers). It was made clear that only individuals from 18 to 60 years old were allowed to participate in the survey. The study was conducted during a three-month period, in the last trimester of 2019 (October to December 2019). It took place in Greece, an EU country that has recently exited a decade of financial distress (2010–2019) (Hazakis, 2021) and made a noteworthy comeback in terms of attracting foreign investment, reducing unemployment and increasing its credibility (Courcoulas and Worrachate, 2021).

A total of 627 questionnaires were received, but 9 questionnaires containing outlier values were rejected (final sample = 618 participants). For identifying outliers, the Mahalanobis distance was computed and a probability test was conducted, based on the Mahalanobis distance and the number of variables (items). All observations (questionnaires) with sig. < 0,001 were considered outliers, according to Barnett and Lewis (1994).

The final sample is deemed to be fairly satisfactory, since it is well above the sample size used by other similar empirical researches (e.g., Yang et al., 2004; Ali et al., 2016; Terblanche, 2018; Syahrial et al., 2017; Walsh et al., 2011; Chen and Lin, 2015; Lee et al., 2008) and comparable to the one used by some other surveys (e.g., Karatepe, 2006; Zhang et al., 2018; Wu and Li, 2018). Taking into consideration the population size (about 6,000,000 Greeks aged between 18-60) and the sample size (618 participants), the margin of error is 3.94% (for a confidence level of 95%), which is considered acceptable. According to Bartlett et al. (2001), an acceptable margin of error (the error the researcher is willing to accept) is between 3% and 5%.

### 4.4 Data analysis

Hypothesis testing was conducted using the “Structural Equation Modeling” (SEM) technique (multivariate analysis) and other similar statistical methods (i.e., Exploratory Factor Analysis and Confirmatory Factors Analysis). SEM can handle complex research models, as the one proposed in this study, in which factors can be both dependent and independent. Also, it offers various measures for testing the overall validity of the proposed model. It can, moreover, examine direct and indirect effects between factors and proposes modification indexes that can help researchers establish additional causal paths (Zhang et al., 2021).

In the present study, covariance-based “Structural Equation Modeling” (CB-SEM) was employed, using the statistical software IBM AMOS 23.0. For examining the proposed research model, the Maximum Likelihood Estimation method was employed. The Covariance Matrix was used as the table of entry and the extraction of the Standardized Completely Solution was requested (Hair et al., 2014; Kelloway, 1998). Moreover, when estimating the structural model (causal paths between factors), mean factor scores were used. These scores were calculated using the mean (average) of the items used for the measurement of each factor. Please note, that this study employed a reflective measurement of its various factors, meaning that measurement items are interchangeable and highly correlated. Hence, using mean scores is a methodologically sound approach (Hair et al., 2014).

### 4.5 Validity and reliability

Content validity was examined before collecting the empirical data. More specifically, a pilot study that included eight consumers was conducted, in order to assess and evaluate the overall effectiveness of the questionnaire and identify potential problems. Additionally, three focus groups were conducted, each with a mixed audience of academics, business consultants and professionals from fields relevant with this study. In average, six individuals participated in each of these groups. The three focus groups discussed the content validity of the questionnaire (e.g., appropriateness, difficulty, clarity, readability, etc.). This dual process (pilot study and focus groups) allowed a number of modifications (mostly, wording issues) that ensured that the questionnaire would be clearly interpreted by the population of this study.

After collecting the appropriate empirical data, the construct validity of the research factors was tested in two consecutive steps. Each of the twelve research factors (constructs) was evaluated: (a) for its unidimensionality and reliability (using the IBM SPSS 25.0 software), (b) for its goodness of fit to the proposed research model (using the IBM AMOS 23.0 software).

(a) The examination of the unidimensionality of each of the research factors was conducted using Explanatory Factor Analysis (EFA) (using Principal Component Analysis and Varimax Rotation). Moreover, the statistical measure ‘Cronbach Alpha’ was used for estimating the reliability of the same factors. More specifically, twelve analyses were conducted, each for every research factor of the proposed research model. On the same vein, the statistical measure ‘Cronbach Alpha’ was also calculated twelve times (one for every factor). Under that context, the following measures were examined (Hair et al., 2014):

⁃ The statistical test of “Kaiser-Mayer-Olkin” (KMO) (values over 0.7 are satisfactory, while values over 0.5 are acceptable).
⁃ The “Bartlett's test of Sphericity” (it should be statistically significant, at the 0.05 level).
⁃ The criterion of “eigenvalue”. Factors whose ‘eigenvalue’ is over one are selected.
⁃ For determining the percent of the total variance that is explained by the proposed factor(s), Total Variance Explained (TVE) was used. TVE should be more than 50%.
⁃ For testing the significance of the items, their factor loadings were examined. A loading over 0.5 is considered significant.
⁃ In order to test the reliability of the various factors, the statistical measure “Cronbach Alpha” was being used. In general, values greater than 0.7 are considered to be valid.

(b) After taking under consideration all the modifications derived from conducting the EFA, the evaluation of the goodness of fit of the research factors was conducted using Confirmatory Factor Analysis (CFA). More specifically, the following measures were examined (Schumacker and Lomax, 2010; Smith and McMillan, 2001):

⁃ Normed Χ^2^ (Χ^2^/degress of freedom): This measure is used because ‘Χ^2^’ seems to be extremely sensitive to sample size. Values between 1 and 3 are desirable, while values between 1 and 5 are acceptable.
⁃ Composite/Construct Reliability (C.R.): Is a measure of internal consistency of a set of items loaded on a latent construct, used complimentary with “Cronbach Alpha”. It should higher than 0.7.
⁃ Average Variance Extracted (A.V.E.): Used to assess the convergent validity of a factor. It should higher than 50%.
⁃ RMSEA (Root Mean Square Error of Approximation): Measures the error of approximation, while taking sample size into account. RMSEA should be less than 0.08 (or 0.1).
⁃ CFI (Comparative Fit Index): Examines the model fit, taking into consideration the discrepancy between the data and the hypothesized model. A value of 0.90 or larger is generally considered to indicate acceptable model fit.
⁃ GFI (Goodness of Fit Index): Measures the fit between the hypothesized model and the observed covariance matrix. A value of 0.90 or larger is generally considered to indicate acceptable model fit.

All tests conducted, produced satisfactory results (see Table 4 below) (CFA factor loadings can be found in the first Appendix of the paper).

**Table 3:** Results of the exploratory factor analysis (EFA).

Factor	Mean	S.D.	ΚΜΟ	Bartlett's Test of Sphericity	Eigen-value	TVE	Cronbach alpha
Physical environment	3.58	0.680	0.817	147.45∗p < 0.001	2.125	53.069	0.817
Interior shop environment & layout	3.27	0.824	0.739	99.92∗p < 0.001	3.066	59.182	0.766
Interaction with the staff	3.85	0.789	0.934	134.91∗p < 0.001	3.354	67.898	0.938
Interaction with other customers	2.95	0.751	0.780	164.47∗p < 0.001	2.614	53.694	0.824
Merchandise value (quality)	3.72	0.759	0.806	113.81∗p < 0.001	3.361	69.802	0.850
Merchandise variety	3.65	0.943	0.676	81.33∗p < 0.001	2.336	63.151	0.790
Customer experience	3.45	0.761	0.863	79.45∗p < 0.001	3.697	65.870	0.894
In-shop emotions	3.48	0.748	0.779	67.15∗p < 0.001	2.679	57.116	0.844
Perceived value	3.51	0.776	0.785	191.72∗p < 0.001	2.455	66.596	0.827
Customer satisfaction	3.84	0.716	0.875	136.94∗p < 0.001	2.891	74.775	0.914
Customer loyalty	3.52	0.881	0.816	97.11∗p < 0.001	3.031	75.360	0.884
Repurchase intention	4.30	0.758	0.834	84.64∗p < 0.001	2.741	82.104	0.913

EFA results dictated to drop out three items from two factors: One item from the factor “Physical environment” and two items from the factor “Customer experience”. After these modifications, the appropriate tests concluded that all the scales used are valid and reliable (see Table 3 for the main results) (EFA factor loadings can be found in Appendix 1).

**Table 4:** Results of the confirmatory factor analysis (CFA).

Factor	Normed Χ^2^	C.R.	A.V.E.	RMSEA	CFI	GFI
Physical environment	2.415	0.890	61.781%	0.062	0.98	0.97
Interior shop environment & layout	1.973	0.861	60.709%	0.058	0.94	0.93
Interaction with the staff	3.061	0.825	54.166%	0.053	0.96	0.91
Interaction with other customers	2.747	0.896	68.274%	0.048	0.97	0.91
Merchandise value (quality)	3.355	0.821	53.522%	0.071	0.99	0.95
Merchandise variety	2.778	0.882	65.323%	0.063	0.97	0.91
Customer experience	2.130	0.919	58.708%	0.067	0.91	0.93
In-shop emotions	3.412	0.861	50.841%	0.081	0.91	0.89
Perceived value	2.266	0.874	63.385%	0.068	0.90	0.96
Customer satisfaction	2.933	0.822	53.790%	0.064	0.97	0.91
Customer loyalty	1.632	0.839	56.748%	0.052	0.93	0.95
Repurchase intention	2.573	0.859	60.458%	0.056	0.97	0.97

Finally, the discriminant validity of the twelve research factors was also examined. Discriminant validity is established when the square root of the “Average Variance Extracted” (A.V.E.) for each individual factor is greater than its correlations with all the other factors of the research model. This methodology (Fornell-Larcker criterion) was proposed by Fornell and Larcker (1981) and is used in many previous studies (e.g., Xu et al., 2014). The corresponding analysis revealed that, with one minor exception, the square roots of the A.V.E. of every factor exceed the inter-factor correlations, indicating that discriminant validity is ensured in this study. Results are presented in Appendix 2.

## 5 Results

### 5.1 Demographic characteristics

The final sample consists mostly of female participants (60.4%) (see Table 5). Most participants belong to the 18–25 (44.2%) or the 36–45 (18.4%) age group. They are well-educated, since 62.9% holds a university degree or have obtained a Postgraduate degree (16%). The majority of the respondents works in the private sector (32%), or is self-employed (16.5%). A large percentage (34.6%) is students (especially those who belong in the age group 18–25) and, consequently, 33.3% reported that they do not gain their own income, but are supported from their family. 16.2% have a monthly income less than 500 Euros and 29.3% a monthly income between 500 and 1,000 Euros. Finally, when it comes to the type of retail store participants had in mind when answering the questionnaire, clothing and shoe stores came first (37.9%) and electronics stores second (23.0%). It is interesting that very few participants had a super market in mind (1.3%, only 8 participants).

**Table 5:** Demographics.

Question	Items	Frequency	Valid Percent
Gender	Male	245	39.6%
	Female	373	60.4%
	***Total***	***618***	***100.0%***
Age groups	18–25 years	273	44.2%
	26–35 years	134	21.7%
	36–45 years	114	18.4%
	46–60 years	97	15.7%
	***Total***	***618***	***100.0%***
Level of education	High school	83	13.4%
	Vocational training	47	7.6%
	University	389	62.9%
	Postgraduate degree	99	16.0%
	***Total***	***618***	***100.0%***
Occupation	Student	214	34.6%
	Unemployed	48	7.8%
	Private sector employee	198	32.0%
	Public sector employee	56	9.1%
	Self-employed	102	16.5%
	***Total***	***618***	***100.0%***
Income	Not working (supported by family)	206	33.3%
	0 - 500 Euros	100	16.2%
	501 - 700 Euros	79	12.8%
	701 - 1000 Euros	102	16.5%
	1001 - 1500 Euros	88	14.2%
	1500 Euros and more	43	7.0%
	***Total***	***618***	***100.0%***
Category of the selected retail store	Department store	52	8.4%
	Clothing and shoes	234	37.9%
	Cosmetics	92	14.9%
	Electronics	142	23.0%
	Home decoration	81	13.1%
	Super market	8	1.3%
	Other	9	1.5%
	***Total***	***618***	***100.0%***

### 5.2 Hypothesis testing

As mention earlier, the examination of the proposed conceptual framework was conducted using the Structural Equation Modeling (SEM) technique. In more detail, the (modified) structural model fitted the data well, while the factors that were included can explain 51% of the variance of the dependent factor “Repurchase Intention”. Also, the variance of all other factors is sufficiently explained (e.g., customer loyalty is explained by 61%).

Additionally, three new paths were added to the structural model (see Table 6) based on the modification indexes of IBM AMOS. The addition of these paths resulted in a structural model with improved fit and explanatory power. Of course, all three paths are rooted in both theory and logic.

**Table 6:** Hypothesis testing.

H	Path			Standardized estimates (r)	p	Result
H1a	Physical environment	**→**	In-shop emotions	0.210	∗∗∗	Accepted
H1b	Physical environment	**→**	Customer experience	0.160	∗∗∗	Accepted
H1c	Physical environment	**→**	Customer satisfaction	-	0.156	Rejected
H2a	Interior shop environment & layout	**→**	In-shop emotions	-	0.254	Rejected
H2b	Interior shop environment & layout	**→**	Customer experience	0.361	∗∗∗	Accepted
H2c	Interior shop environment & layout	**→**	Customer satisfaction	-	0.261	Rejected
H3a	Interaction with the staff	**→**	In-shop emotions	-	0.111	Rejected
H3b	Interaction with the staff	**→**	Customer experience	0.148	∗∗∗	Accepted
H3c	Interaction with the staff	**→**	Customer satisfaction	0.115	∗∗∗	Accepted
H4a	Interaction with other customers	**→**	In-shop emotions	-	0.069	Rejected
H4b	Interaction with other customers	**→**	Customer experience	0.121	∗∗∗	Accepted
H4c	Interaction with other customers	**→**	Customer satisfaction	-	0.097	Rejected
H5a	Merchandise value (quality)	**→**	In-shop emotions	0.155	∗∗∗	Accepted
H5b	Merchandise value (quality)	**→**	Customer experience	0.153	∗∗∗	Accepted
H5c	Merchandise value (quality)	**→**	Customer satisfaction	0.209	∗∗∗	Accepted
H6a	Merchandise variety	**→**	In-shop emotions	-	0.211	Rejected
H6b	Merchandise variety	**→**	Customer experience	-	0.126	Rejected
H6c	Merchandise variety	**→**	Customer satisfaction	0.080	∗∗∗	Accepted
H7a	Customer experience	**→**	Customer satisfaction	0.128	∗∗∗	Accepted
H7b	Customer experience	**→**	Customer loyalty	0.128	∗∗∗	Accepted
H7c	Customer experience	**→**	Perceived value	0.219	∗∗∗	Accepted
H7d	Customer experience	**→**	Repurchase intention	-	0.331	Rejected
H8a	In-shop emotions	**→**	Customer satisfaction	0.396	∗∗∗	Accepted
H8b	In-shop emotions	**→**	Customer loyalty	0.192	∗∗∗	Accepted
H8c	In-shop emotions	**→**	Perceived value	-	0.127	Rejected
H8d	In-shop emotions	**→**	Repurchase intention	-	0.099	Rejected
H9a	Perceived value	**→**	Customer satisfaction	0.143	∗∗∗	Accepted
H9b	Perceived value	**→**	Customer loyalty	0.333	∗∗∗	Accepted
H9c	Perceived value	**→**	Repurchase intention	0.159	∗∗∗	Accepted
H10a	Customer satisfaction	**→**	Customer loyalty	0.202	∗∗∗	Accepted
H10b	Customer satisfaction	**→**	Repurchase intention	0.333	∗∗∗	Accepted
H11	Customer loyalty	**→**	Repurchase intention	0.335	∗∗∗	Accepted
***Newly proposed causal paths***						
NP1	Merchandise value (quality)	**→**	Customer loyalty	0.144	∗∗∗	New path
NP2	Merchandise value (quality)	**→**	Perceived value	0.431	∗∗∗	New path
NP3	Customer experience	**→**	In-shop emotions	0.544	∗∗∗	New path

∗∗∗p < 0.001, ∗∗p < 0.01, ∗p < 0.05.

Firstly, the relationship between customer experience and in-shop emotions (NP3) was found to be quite strong (r = 0**.**544). It is only reasonable that the customer interaction inside a store will have an impact on the emotions that are generated (Goić et al., 2021). Despite the fact that this relationship has never been investigated before, the present study argues that when a retail store engages, stimulates and intrigues customers, their overall emotions towards that store are becoming increasingly positive. Kuppelwieser (2021) offers a framework that summarizes the main challenges for customer experience in the modern retail environment. In that context, the role of emotions is thoroughly discussed. The two concepts of customer experience and in-shop emotions seem to be highly correlated (De Keyser et al., 2020; Verhulst et al., 2020).

Secondly, the relationship between merchandise value and perceived (store) value (NP2, r = 0.431) is also quite reasonable, since stores that offer reliable products of high perceived quality are considered more attractive and valuable in comparison with other alternatives. For example, Ligas and Chaudhuri (2012) found that merchandise value has a significant impact on perceived store usefulness and hedonic store value. Both of these factors are associated with the value a customer receives from a store. When examining the two antecedents of perceived value, it seems that merchandise value has the highest impact, meaning that customers are really interested about the quality of the products, their proper functionality and the prices that offer value for money.

Finally, the third causal path added to the proposed model (NP1) argues that merchandise value has an impact on customer loyalty. This path is not as strong as the previous two, but it is still statistically significant (r = 0.144, p < 0.001). It seems that when retail customers perceive that a store offers valuable products, the level of their loyalty is increased (Terblanche and Boshoff, 2006). Ligas and Chaudhuri (2012) concluded that merchandise value affects the willingness of customers to pay a higher price. On the same vein, Chaudhuri and Ligas (2009) argue that, based on cognitive psychology, merchandise value is related to both repurchase and attitudinal loyalty. Firstly, merchandise value can lead to a purely transactional exchange (i.e., repurchase loyalty, but without commitment); secondly, it can lead to a more enduring relationship that incorporates some kind of bond or connectedness with the retail store (i.e., attitudinal loyalty with commitment) (Aurier and de Lanauze, 2011; Chaudhuri and Ligas, 2009; Wierich and Zielke, 2014).

**Figure 2:**
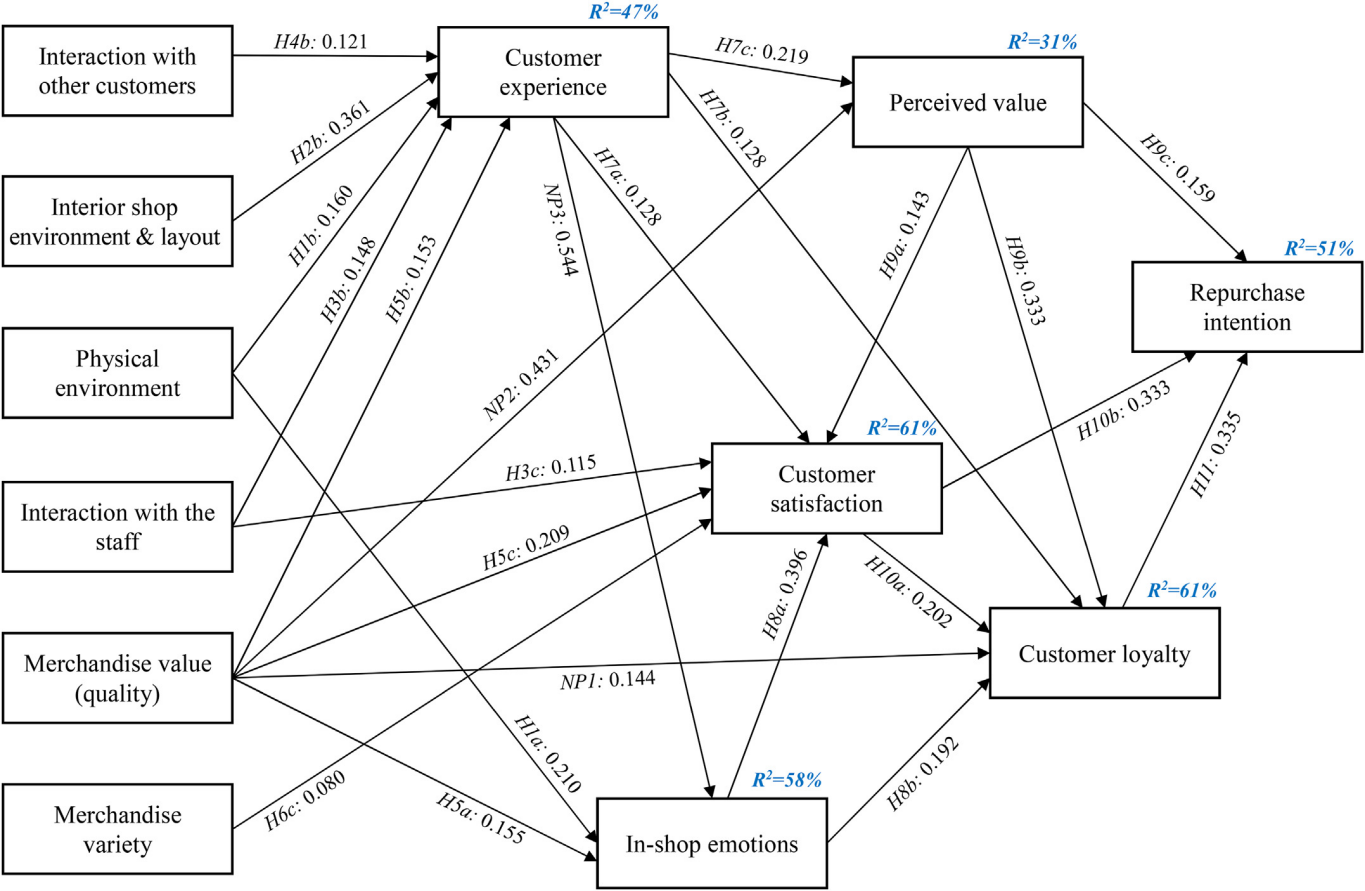
The modified research model (all paths are statistically significant/standardized estimates are included).

Table 6 presents the results of the Hypothesis testing process, along with the path coefficients of every supported relationship (statistically insignificant paths have been removed from the model and they are not reported in Table 6). Additionally, Table 7 includes the direct, indirect and total effects between research factors, while Figure 2 demonstrates the overall structural model, along with the extracted path coefficients and the adjusted R^2^ scores.

**Table 7:** Total (T), Direct (D) and Indirect (I) effects (standardized estimates) between research factors.

		Physical environment	Interior shop environment & layout	Interaction with the staff	Interaction with other customers	Merchandise value (quality)	Merchandise variety	Customer experience	In-shop emotions	Perceived Value	Customer Satisfaction	Customer loyalty
Customer experience	D	0.160∗∗∗	0.361∗∗∗	0.148∗∗∗	0.121∗∗	0.153∗∗∗						
	I											
	T	0.160∗∗∗	0.361∗∗∗	0.148∗∗∗	0.121∗∗	0.153∗∗∗						
In-shop emotions	D	0.210∗∗∗				0.155∗∗∗		0.544∗∗∗				
	I	0.087∗∗∗	0.196∗∗∗	0.080∗∗∗	0.066∗∗	0.083∗∗∗						
	T	0.297∗∗∗	0.196∗∗∗	0.080∗∗∗	0.066∗∗	0.238∗∗∗		0.544∗∗∗				
Perceived value	D					0.431∗∗∗		0.219∗∗∗				
	I	0.035∗∗∗	0.079∗∗∗	0.032∗∗∗	0.027∗∗	0.033∗∗∗						
	T	0.035∗∗∗	0.079∗∗∗	0.032∗∗∗	0.027∗∗	0.464∗∗∗		0.219∗∗∗				
Customer satisfaction	D			0.115∗∗∗		0.209∗∗∗	0.080∗∗	0.128∗	0.396∗∗∗	0.143∗∗∗		
	I	0.143∗∗∗	0.135∗∗∗	0.055∗∗∗	0.046∗∗	0.180∗∗∗		0.247∗∗∗				
	T	0.143∗∗∗	0.135∗∗∗	0.171∗∗∗	0.046∗∗	0.389∗∗∗	0.080∗∗	0.375∗∗	0.396∗∗∗	0.143∗∗∗		
Customer loyalty	D					0.144∗∗∗		0.128∗∗∗	0.192∗∗∗	0.333∗∗∗	0.202∗∗∗	
	I	0.118∗∗∗	0.137∗∗∗	0.079∗∗∗	0.046∗∗	0.298∗∗∗	0.016∗∗	0.253∗∗∗	0.080∗∗∗	0.029∗∗∗		
	T	0.118∗∗∗	0.137∗∗∗	0.079∗∗∗	0.046∗∗	0.442∗∗∗	0.016∗∗	0.381∗∗∗	0.272∗∗∗	0.361∗∗∗	0.202∗∗∗	
Repurchase intention	D									0.159∗∗∗	0.333∗∗∗	0.335∗∗∗
	I	0.093∗∗∗	0.104∗∗∗	0.089∗∗∗	0.035∗∗	0.352∗∗∗	0.032∗∗	0.287∗∗∗	0.223∗∗∗	0.169∗∗∗	0.068∗∗∗	
	T	0.093∗∗∗	0.104∗∗∗	0.089∗∗∗	0.035∗∗	0.352∗∗∗	0.032∗∗	0.287∗∗∗	0.223∗∗∗	0.328∗∗∗	0.400∗∗∗	0.335∗∗∗

∗∗∗p < 0.001, ∗∗p < 0.01, ∗p < 0.05 (p values are calculated via the bootstrap method).

More analytically, to evaluate the fit of the modified model, the chi-square value (X^2^ = 141.33 with 30 degrees of freedom) and the p-value (p < 0.001) were estimated. These values indicate a poor fit of the data to the overall model. However, the sensitivity of the X^2^ statistic to the sample size (n = 618), necessitates the adoption of other supplementary measures for evaluating the overall model (Smith and McMillan, 2001), such as the “Normed-X^2^” index (4.711), the RSMEA index (0.093), the RMR (Root Mean square Residual) index (0.061), the CFI (0.690) and the GFI (0.954). These measures, when examined collectively, indicate a satisfactory fit of the modified model.

## 6 Analysis of the results

### 6.1 General remarks

Overall, in accordance with the existing literature, empirical results support most of the research hypotheses (see Table 6). At the same time, some new relationships emerged, further enhancing the importance of the modified research model.

The predictive power of the revised research model (see Figure 2) is very satisfactory. More specifically, the variance of its five mediating factors (that capture various aspects of consumer behavior) is sufficiently explained: (a) Customer experience (by 47%), (b) In-shop emotions (by 58%), (c) Perceived value (by 31%), (d) Customer satisfaction (by 61%), (e) Customer loyalty (by 61%). Moreover, the variance of the dependent factor of this study, Repurchase intention, is explained by 51%. Therefore, the proposed research model can successfully explain the variance of its main dependent factor.

Results indicate that the six independent factors included in this study, which measure the in-store customer shopping experience, can successfully predict the variance of various factors capturing aspects of consumer behavior. These results further enhance the findings of Terblanche (2018), who found a positive impact of in-store customer shopping experience on customer satisfaction. The present study advocates that in-store shopping experience has a much wider effect, also affecting customer experience, perceived value, customer loyalty and customer satisfaction.

Moreover, empirical results highlight the significant role of all five mediators used in this study (in-shop emotions, customer experience, perceived value, customer loyalty, customer satisfaction) in predicting repurchase consumer intentions. Overall, independents (aspects of in-store customer shopping experience) and mediators (aspects of consumer behavior) act as a bundle of practices: Independent factors affect the mediators; the mediators affect one another and cumulatively enhance repurchase intentions.

### 6.2 Causal effects between research factors

The results support almost all the initial hypotheses. More specifically, out of the initial 32 hypotheses, only hypotheses 3a, 4a, 4c, 6a, 6b and 8c are rejected, while 1c, 2a, 2c, 7d and 8d are accepted based on the examination of the results concerning their indirect relationships.

Table 7 analytically presents the direct, indirect and total effects between research factors. Its careful examination offers room for interesting observations. In that direction, it is concluded that merchandise value (quality) is the most influential of the independent factors of the study. It strongly affects customer experience (0.153), in-shop emotions (0.238), perceived value (0.464), customer satisfaction (0.389), customer loyalty (0.442), and repurchase intention (0.352). It directly influences all five mediators (aspects of consumer behavior). Moreover, it has an indirect effect on four of them, while it also indirectly affects repurchase intention. On average, its impact on the five mediators and the dependent factor is 0.340. The other two most influential independent factors are interior shop environment and layout (average impact on the five mediators and the dependent factor = 0.169), and physical environment (0.141).

Thus, the assumption that merchandise quality increases repurchase intention is empirically supported. Interestingly enough, the present study found out that this relationship is not direct, but only indirect. This finding, actually, indicates that merchandise quality functions supportively, enhancing other factors that directly affect repurchase intention.

When examining the role of customer experience, it is concluded that it directly influences the emotions evoked while being inside a store (0.544). This observation appears to be in line with the findings of the international literature, according to which customer experience stimulates emotions (Chen and Lin, 2015). For some, the emotions evoked while inside the store constitute an integral part of customer experience. Previous studies have not thoroughly studied customer experience and emotions while being in a shop. They, rather, focused on customer satisfaction, as being the only dependent factor of customer-shop interaction. This study has attempted to investigate emotions that may be evoked during the presence of a buyer inside a shop.

Both customer experience and in-shop emotions significantly affect the other mediating factors (aspects of consumer behavior). More specifically, customer experience directly affects perceived value (0.219), customer satisfaction (0.128) and customer loyalty (0.128), while in-shop emotions directly affect customer satisfaction (0.396) and customer loyalty (0.192). Both of them only indirectly affect repurchase intention (0.287 and 0.223, respectively). Hence, it could be assumed that enhanced customer experience indirectly reinforces repurchase intention, firstly by boosting other factors, such as customer loyalty and satisfaction. The importance of the experience a customer has inside a store is, thus, underlined and the need to create engaging experiences is emphasized.

Moreover, customer satisfaction and customer loyalty have a direct effect on repurchase intention (0.333 and 0.335, respectively), something which is also in line with the literature (e.g., Terblanche, 2018). Empirical results highlight customer satisfaction as the determinant factor that leads to enhanced customer loyalty and repurchase intention. Once customers become loyal to a specific store, it is difficult for it to be anything but their first choice, even if it is the source of occasional disappointment. Loyal customers will always give a second chance to shops they are generally happy with (Joireman et al., 2013).

Finally, it was found that perceived value also affects repurchase intention, both directly (0.159) and indirectly (0.169). Among the three factors that have a direct effect on repurchase intention, the direct effect of perceived value is the lowest (customer satisfaction: 0.333, customer loyalty: 0.335). Despite that, its total effect, as seen on Table 7, is almost at the same level with the others two factors (perceived value: 0.328 customer satisfaction: 0.400, customer loyalty: 0.335). This finding underlines its overall significance and urges retail stores to focus on the value they offer to their customers.

## 7 Conclusions

The goal of this study was to empirically investigate the factors affecting the behavior of retail consumers and their intention to repurchase from a retail store. In order to achieve that, an original research model was developed and empirically tested in a sample of 618 retail store customers. This model adopts a three-dimensional approach, including the following set of factors: (a) Independent factors that capture the “in-store customer shopping experience”, (b) Mediating factors that capture various aspects of consumer behavior, (c) one dependent factor, namely repurchase intention. Independent factors are considered the antecedents of consumer behavior, while mediating factors mediate the effect of the antecedents on repurchase intention (antecedents → mediators → repurchase intention). This approach is unique in the international literature of the field. Also, the study focuses on retail stores in general, offering wider generalizability of its results. Finally, it includes an environmental psychology perspective, examining customer in-store emotions and customer experience, something that few studies have attempted in the retail context.

The nature and intensity of the causal relations examined in this study are of great interest, both for retailers, as well as marketing executives. According to the empirical findings, the four factors with the strongest total effect on repurchase intention are: (a) customer satisfaction; (b) merchandise value (quality); (c) customer loyalty and (d) perceived value.

The literature underlines the importance of customer trust and the positive behavioral intentions it invokes (Han et al., 2008; Hume and Mort, 2010; Loureiro et al., 2014), while the role of customer satisfaction and emotion in the development of loyalty has also been stressed out. Loyalty, in turn, has a significant impact on behavior and consumer attitude (Shkoukani et al., 2017). In the marketing community, customer loyalty has long stood as the most important objective (Reichheld and Schefter, 2000). Many empirical studies have noted that the most effective and efficient mean for developing loyal customers is the firm's ability to satisfy their needs (Lee et al., 2001; Oliver, 1999) by offering superior value (Parasuraman and Grewal, 2000). In agreement with Ali et al. (2016), this study found that customer satisfaction has a positive impact on customer loyalty.

The positive relationship between Interaction with the staff, In-shop emotions, Merchandise value (quality), merchandise variety, on the one hand, and customer satisfaction, on the other hand, was also verified. Moreover, it was found out that the same factors are indirectly related with customer repurchase intention. Satisfaction influences repurchase intention both directly and indirectly, while merchandise value plays a strengthening role, having an effect on the factors that directly affect repurchase intention. Under that context, merchandise value emerges as the most crucial of the six independent factors that capture the “in-store customer shopping experience”.

The fact that very few of the proposed initial hypotheses have been rejected by the empirical data signifies the validity, as well as the reliability of the proposed research model. Merchandise value has a particularly significant effect on repurchase intention, although it is not directly related to it. It reinforces various factors, such as customer satisfaction and customer loyalty and exhibits a very significant indirect effect on repurchase intention. Thus, the higher the quality of the products offered by an enterprise, the more satisfied its customers will be. Satisfaction, in turn, leads also to loyalty.

It is also worth mentioning that customer loyalty is further reinforced by a fairly large number of factors. These include perceived value, in-shop emotions, merchandise value, customer experience and customer satisfaction. In order, therefore, to increase customer repurchase intention and future profits, organizations must, firstly, focus on the reinforcement of customer loyalty.

In synopsis, the present study draws the following main conclusions: (a) Its three-dimensional research model successfully predicts the variance in repurchase intentions. Future studies are urged to follow the same approach; (b) Merchandise value is the most significant aspect of the in-store customer shopping experience, since it has a strong indirect effect on repurchase intentions; (c) In-shop emotions and customer satisfaction are significant determinants of repurchase intention. The former have a strong effect on the latter, while they both indirectly enhance the intention of customers to buy from the same physical store; (d) All aspects of consumer behavior (customer experience, in-shop emotions, perceived value, customer satisfaction, customer loyalty) are intercorrelated and enhance repurchase intentions, meaning that retailers should focus on their collective enhancement.

### 7.1 Managerial implications

Initially, the quality of the service provided to customers must be equal to their expectations. In order to achieve this, an excellent organization of the premises is required, in order for products to be visible and for customers not to waste their time aimlessly searching for them. The space must, therefore, be clean, well-organized and with pleasant colors (walls, furniture, decorations) and scents (e.g., use of fresheners).

Employees must also be fully informed on the duties of their post, so that they may assist customers offering them relevant and valuable information. They must be friendly, polite and willing to service them. Customers must feel comfortable, so the appropriate type of competent personnel is required. A “festive ambiance” inside the shop should be created, in order to make customer experience in the shop more exciting. This may entail various “happenings”, which should take place in the shop during the day.

This research also ascertained the existence of a significant positive effect of merchandise value on repurchase intention. The marketing executives and shop managers must, therefore, pay particular attention to the quality of their merchandise. Consumers many times emphasize on quality, not necessarily being equally interested in the cost of the product/service. There must be a good quality-price (value for money) equilibrium (perceived value), since prohibitively expensive products may potentially avert several potential buyers from choosing them.

Customers must perceive and understand if they were treated fairly and properly by the firm, as it is reflected on their expenses, the quality of the products they receive, as well as their interaction with the staff. Customers must never feel that they have “wasted” their time and money, since this might indicate that the firm lost a client who will search elsewhere for a more suitable product offering.

It is obvious that in order to influence repurchase intention, firms must create dedicated and loyal customers. For a firm to succeed this, it must keep them almost always satisfied. It was observed that satisfaction has many folds and can be expressed in many ways. The quality of the products/services is equally important for increasing loyalty and repurchases intention. When customers remain satisfied from the quality of the merchandise they buy, they have no reason to seek for some other stores to buy the same type of goods/services.

## Declarations

### Author contribution statement

Prodromos Chatzoglou: Conceived and designed the experiments; Contributed reagents, materials, analysis tools or data; Wrote the paper.

Dimitrios Chatzoudes: Conceived and designed the experiments; Performed the experiments; Analyzed and interpreted the data; Wrote the paper.

Athina Savvidou: Performed the experiments; Wrote the paper.

Thomas Fotiadis; Pavlos Delias: Analyzed and interpreted the data; Wrote the paper.

### Funding statement

No funding was received for this study.

### Data availability statement

Data will be made available on request.

### Declaration of interest's statement

The authors declare the following conflict of interests: One of the authors is a member of the Editorial team of the journal (Section Editor for the B&E section).

### Additional information

No additional information is available for this paper.

## Figures and Tables

**Table:** **Physical environment** (Ali et al., 2016):

PE1. The store's lighting is appropriate.	(EFA_loading_ = 0.879, CFA_loading_ = 0.801)
PE2. The store's temperature is comfortable.	(EFA_loading_ = 0.793, CFA_loading_ = 0.748)
PE3. The store's environment is clean.	(EFA_loading_ = 0.823, CFA_loading_ = 0.789)
PE4. The store's architecture and setting is impressive.	(EFA_loading_ = 0.744, CFA_loading_ = 0.756)
PE5. The colors within the store are complementary and coordinating.	(EFA_loading_ = 0.812, CFA_loading_ = 0.833)

**Table:** **Interior shop environment & layout** (Terblanche, 2018):

ISEL1. The store has attractive product and promotional displays.	(EFA_loading_ = 0.912, CFA_loading_ = 0.789)
ISEL2. The store has attractive décor.	(EFA_loading_ = 0.863, CFA_loading_ = 0.759)
ISEL3. The store has attractive in-store physical facilities (check-out counters, shelves, etc.).	(EFA_loading_ = 0.867, CFA_loading_ = 0.769)
ISEL4. The store has attractive materials associated with its service (shopping bags, brochures, etc).	(EFA_loading_ = 0.901, CFA_loading_ = 0.799)

**Table:** **Interaction with the staff** (Ali et al., 2016):

IWTS1. The staff provides a thorough and satisfactory service.	(EFA_loading_ = 0.813, CFA_loading_ = 0.777)
IWTS2. The staff is reliable.	(EFA_loading_ = 0.763, CFA_loading_ = 0.679)
IWTS3. The staff is professional.	(EFA_loading_ = 0.783, CFA_loading_ = 0.723)
IWTS4. The staff has a good knowledge of the products.	(EFA_loading_ = 0.861, CFA_loading_ = 0.761)

**Table:** **Interaction with other customers** (Ali et al., 2016):

IWOC1. Other customers (in this store) are not loud.	(EFA_loading_ = 0.901, CFA_loading_ = 0.833)
IWOC2. Other customers (in this store) behave nicely.	(EFA_loading_ = 0.903, CFA_loading_ = 0.830)
IWOC3. Other customers (in this store) are not problematic.	(EFA_loading_ = 0.843, CFA_loading_ = 0.861)
IWOC4. Other customers (in this store) do not create disturbance.	(EFA_loading_ = 0.863, CFA_loading_ = 0.779)

**Table:** **Merchandise value (quality)** (Terblanche, 2018):

MVQ1. The products sold in this store are reliable.	(EFA_loading_ = 0.833, CFA_loading_ = 0.738)
MVQ2. The products sold in this store are free from defects and flaws.	(EFA_loading_ = 0.748, CFA_loading_ = 0.679)
MVQ3. The products sold in this store represent good value.	(EFA_loading_ = 0.769, CFA_loading_ = 0.716)
MVQ4. The products sold in this store have good quality.	(EFA_loading_ = 0.864, CFA_loading_ = 0.789)

**Table:** **Merchandise variety** (Terblanche, 2018):

MV1. The store offers a good selection of well-known brands.	(EFA_loading_ = 0.872, CFA_loading_ = 0.757)
MV2. The store offers a wide variety of products.	(EFA_loading_ = 0.907, CFA_loading_ = 0.824)
MV3. The store covers most of my consumer needs.	(EFA_loading_ = 0.749, CFA_loading_ = 0.741)
MV4. All the known brands are available in this store.	(EFA_loading_ = 0.835, CFA_loading_ = 0.901)

**Table:** **Customer experience** (Chen and Lin, 2015):

CE1. The store tries to engage my senses.	(EFA_loading_ = 0.789, CFA_loading_ = 0.751)
CE2. Visiting this store is perceptually interesting.	(EFA_loading_ = 0.746, CFA_loading_ = 0.703)
CE3. The store tries to put me in a certain mood.	(EFA_loading_ = 0.823, CFA_loading_ = 0.746)
CE4. The store makes me respond in an emotional manner.	(EFA_loading_ = 0.726, CFA_loading_ = 0.791)
CE5. The store tries to intrigue me.	(EFA_loading_ = 0.863, CFA_loading_ = 0.831)
CE6. The store stimulates my curiosity.	(EFA_loading_ = 0.741, CFA_loading_ = 0.766)
CE7. The store tries to make me think about my lifestyle.	(EFA_loading_ = 0.693, CFA_loading_ = 0.801)
CE8. I can relate to other people through this store.	(EFA_loading_ = 0.827, CFA_loading_ = 0.733)

**Table:** **In-shop emotions** (Lee et al., 2008):

ISE1. When I am in this store, I feel happy.	(EFA_loading_ = 0.735, CFA_loading_ = 0.647)
ISE2. When I am in this store, I feel excited.	(EFA_loading_ = 0.746, CFA_loading_ = 0.726)
ISE3. When I am in this store, I feel energetic.	(EFA_loading_ = 0.803, CFA_loading_ = 0.699)
ISE4. When I am in this store, I feel bored (R).	(EFA_loading_ = 0.697, CFA_loading_ = 0.753)
ISE5. When I am in this store, I feel angry (R).	(EFA_loading_ = 0.710, CFA_loading_ = 0.744)
ISE6. When I am in this store, I feel annoyed (R).	(EFA_loading_ = 0.739, CFA_loading_ = 0.704)

**Table:** **Perceived value** (Yang and Peterson, 2004):

PV1. Compared to alternative stores, this store offers attractive product costs.	(EFA_loading_ = 0.911, CFA_loading_ = 0.756)
PV2. Compared to alternative stores, this store charges me fairly for similar products.	(EFA_loading_ = 0.864, CFA_loading_ = 0.812)
PV3. Compared to alternative stores, this store provides better in-store service.	(EFA_loading_ = 0.791, CFA_loading_ = 0.793)
PV4. Comparing what I pay to what I might get from other competitive stores, I think that this store provides me with good value.	(EFA_loading_ = 0.895, CFA_loading_ = 0.822)

**Table:** **Customer satisfaction** (Ali et al., 2016; Terblanche, 2018):

CS1. The store does a good job with the satisfaction of my needs.	(EFA_loading_ = 0.836, CFA_loading_ = 0.721)
CS2. I am very satisfied with the products provided by the store.	(EFA_loading_ = 0.741, CFA_loading_ = 0.647)
CS3. The store covers my needs.	(EFA_loading_ = 0.887, CFA_loading_ = 0.756)
CS4. The experience that I have had with the store has been satisfactory.	(EFA_loading_ = 0.806, CFA_loading_ = 0.801)

**Table:** **Customer loyalty** (Zhang et al., 2018; Wu and Li 2018):

CL1. I speak favorably about this store to others.	(EFA_loading_ = 0.923, CFA_loading_ = 0.846)
CL2. I speak favorably about this store to others.	(EFA_loading_ = 0.927, CFA_loading_ = 0.763)
CL3. I encourage my friends and relatives to visit that store.	(EFA_loading_ = 0.773, CFA_loading_ = 0.656)
CL4. This store is always my first choice.	(EFA_loading_ = 0.864, CFA_loading_ = 0.736)

**Table:** **Repurchase intention** (Terblanche, 2018):

RI1. I intend to shop at this store again in the future.	(EFA_loading_ = 0.991, CFA_loading_ = 0.756)
RI2. I am likely to visit this store again in the future.	(EFA_loading_ = 0.992, CFA_loading_ = 0.811)
RI3. I will probably use the products (and associated services) of this store in the future.	(EFA_loading_ = 0.846, CFA_loading_ = 0.761)
RI4. I will shop more at this store in the future.	(EFA_loading_ = 0.896, CFA_loading_ = 0.781)

**Table:** 

Square root of the A.V.E.		0,786	0,779	0,736	0,826	0,732	0,808	0,766	0,713	0,796	0,733	0,753	0,778
		(1)	(2)	(3)	(4)	(5)	(6)	(7)	(8)	(9)	(10)	(11)	(12)
(1) Physical environment	*r*	1											
(2) Interior shop environment & layout	*r*	0.580∗∗∗	1										
(3) Interaction with the staff	*r*	0.474∗∗∗	0.327∗∗∗	1									
(4) Interaction with other customers	*r*	0.514∗∗∗	0.323∗∗∗	0.447∗∗∗	1								
(5) Merchandise value (quality)	*r*	0.427∗∗∗	0.318∗∗∗	0.457∗∗∗	0.430∗∗∗	1							
(6) Merchandise variety	*r*	0.360∗∗∗	0.342∗∗∗	0.308∗∗∗	0.312∗∗∗	0.479∗∗∗	1						
(7) Customer experience	*r*	0.563∗∗∗	0.588∗∗∗	0.461∗∗∗	0.448∗∗∗	0.456∗∗∗	0.382∗∗∗	1					
(8) In-shop emotions	*r*	0.579∗∗∗	0.472∗∗∗	0.419∗∗∗	0.456∗∗∗	0.496∗∗∗	0.323∗∗∗	0.733∗∗∗	1				
(9) Perceived value	*r*	0.314∗∗∗	0.243∗∗∗	0.350∗∗∗	0.277∗∗∗	0.546∗∗∗	0.285∗∗∗	0.423∗∗∗	0.380∗∗∗	1			
(10) Customer satisfaction	*r*	0.553∗∗∗	0.403∗∗∗	0.499∗∗∗	0.398∗∗∗	0.622∗∗∗	0.424∗∗∗	0.644∗∗∗	0.707∗∗∗	0.514∗∗∗	1		
(11) Customer loyalty	*r*	0.459∗∗∗	0.323∗∗∗	0.406∗∗∗	0.427∗∗∗	0.606∗∗∗	0.345∗∗∗	0.604∗∗∗	0.626∗∗∗	0.640∗∗∗	0.682∗∗∗	1	
(12) Repurchase intention	*r*	0.369∗∗∗	0.257∗∗∗	0.295∗∗∗	0.250∗∗∗	0.543∗∗∗	0.274∗∗∗	0.442∗∗∗	0.527∗∗∗	0.549∗∗∗	0.652∗∗∗	0.671∗∗∗	1

∗∗∗p < 0.001.
